# Genome-wide transcriptional and physiological responses to drought stress in leaves and roots of two willow genotypes

**DOI:** 10.1186/s12870-015-0630-2

**Published:** 2015-10-12

**Authors:** Pascal Pucholt, Per Sjödin, Martin Weih, Ann Christin Rönnberg-Wästljung, Sofia Berlin

**Affiliations:** Department of Plant Biology, Uppsala BioCenter, Linnean Centre for Plant Biology, Swedish University of Agricultural Sciences, SE-750 07 Uppsala, Sweden; Department of Evolutionary Biology, Evolutionary Biology Centre, Uppsala University, SE-752 36 Uppsala, Sweden; Department of Crop Production Ecology, Linnean Center for Plant Biology, Swedish University of Agricultural Sciences, SE-750 07 Uppsala, Sweden

**Keywords:** Drought stress, Response to drought, RNA-seq, *De novo* assembly, *Salix*, Differentially expressed genes, Candidate genes

## Abstract

**Background:**

Drought is a major environmental stress that can have severe impacts on plant productivity and survival. Understanding molecular mechanisms of drought responses is crucial in order to breed for drought adapted plant cultivars. The aim of the present study was to investigate phenotypic and transcriptional drought responses in two willow genotypes (520 and 592) originating from an experimental cross between *S*. *viminalis × *(*S. viminalis × S. schwerinii*). Willows are woody perennials in the Salicaceae plant family that are grown as bioenergy crops worldwide.

**Methods:**

An experiment was conducted where plants were exposed to drought and different eco-physiological parameters were assessed. RNA-seq data was furthermore generated with the Illumina technology from root tips and leaves from plants grown in drought and well-watered (WW) conditions. The RNA-seq data was assembled de novo with the Trinity assembler to create a reference gene set to which the reads were mapped in order to obtain differentially expressed genes (DEGs) between the drought and WW conditions. To investigate molecular mechanisms involved in the drought response, GO enrichment analyses were conducted. Candidate genes with a putative function in the drought response were also identified.

**Results:**

A total of 52,599 gene models were obtained and after filtering on gene expression (FPKM ≥ 1), 35,733 gene models remained, of which 24,421 contained open reading frames. A total of 5,112 unique DEGs were identified between drought and WW conditions, of which the majority were found in the root tips. Phenotypically, genotype 592 displayed less growth reduction in response to drought compared to genotype 520. At the transcriptional level, genotype 520 displayed a greater response in the leaves as more DEGs were found in genotype 520 compared to genotype 592. In contrast, the transcriptional responses in the root tips were rather similar between the two genotypes. A core set of candidate genes encoding proteins with a putative function in drought response was identified, for example MYBs and bZIPs as well as chlorophyll a/b binding proteins.

**Discussion:**

We found substantial differences in drought responses between the genotypes, both at the phenotypic and transcriptional levels. In addition to the genotypic variation in several traits, we also found indications for genotypic variation in trait plasticity, which could play a role in drought adaptation. Furthermore, the two genotypes displayed overall similar transcriptional responses in the root tips, but more variation in the leaves. It is thus possible that the observed phenotypic differences could be a result of transcriptional differences mostly at the leaf level.

**Conclusions:**

This study has contributed to a better general understanding of drought responses in woody plants, specifically in willows, and has implications for breeding research towards more drought adapted plants.

**Electronic supplementary material:**

The online version of this article (doi:10.1186/s12870-015-0630-2) contains supplementary material, which is available to authorized users.

## Background

Drought is a major environmental stress that can have severe impacts on plant productivity and survival. Upon drought, plants will perform an array of complex responses, involving molecular, biochemical, physiological and morphological changes [1, 2]. As a result of adaptation to environmental conditions, for example water availability and drought, various drought avoidance and tolerance mechanisms have evolved. These mechanisms include whole-plant changes such as shoot-root allocation, growth rate, leaf morphology, leaf abscission, stomatal conductance and photosynthetic rate, as well as molecular changes underpinned by remodelling of the transcriptome that may include upregulation of stress signalling, transcription factors and defence processes [1, 3]. Due to the availability of the *Populus trichocarpa* genome sequence [4], transcriptional responses to abiotic stresses in woody plants have primarily been studied in various poplar species. Comparisons of gene expression levels between samples from different tissues, time points and treatments has been performed using microarrays [5–7] or more recently massively parallel sequencing of total RNA, i.e. RNA-seq [8–10]. As a result, a wealth of information on genes and processes involved in the molecular mechanisms of drought responses in different poplar species is available. These studies have revealed large variation in transcriptional drought responses between different species. For example, in the leaves of *P. trichocarpa*, 5689 genes were differentially expressed between drought and control conditions [10], while in the leaves of *P. balsamifera* only 98 probe sets displayed increased transcript abundance under drought [11]. Other studies have revealed variation in drought responses between populations [12–14] and even between genotypes of the same species [11]. These observed differences could originate from genetic diversification as a result of adaption to different drought conditions [15]. Such diversifying selection should be visible as genetic variation in important genes involved in drought response. Thus, identifying genetic variation associated with variation in phenotypic responses, could provide valuable insight into the genetic basis of drought adaptation. The ultimate goal would be to pinpoint genetic variants that are associated with phenotypic responses to drought that could then be used in marker assisted selection to produce cultivars better adapted to drought conditions. In order to do so, there is an urgent need for information on phenotypic and transcriptional responses from species other than model-species. In this work we have examined and compared drought responses in willow genotypes (genus *Salix*, the sister genus to *Populus*) that, like poplars, are woody perennials in the Salicaceae family. Species from both genera have for a long time been grown as bioenergy crops worldwide and there are active breeding programs for developing new high performing varieties [16, 17]. Willows are particularly suitable for cultivations in regions with good water supply such as most of Northern Europe [18]. However, considering that willows appear to be prone to drought stress [19, 20], drought tolerant varieties are desired for cultivations in southern Europe [21]. For the purpose of bioenergy, *Salix viminalis*, *S. dascyclados* and *S. schwerinii* and their hybrids are the mostly used species in Europe as they display rapid growth and high biomass yields [16, 22]. There is relatively high genetic diversity in natural populations of willows [23], which have proven useful for the identification of quantitative trait loci (QTLs) of different traits such as frost and rust resistance and phenology [24–28], growth, water-use efficiency and drought tolerance [19, 29, 30].

The aim of the present study was to investigate phenotypic and transcriptional drought responses in two willow genotypes originating from an experimental cross between *S. viminalis* × (*S. viminalis* × *S. schwerinii*) [31]. In order to achieve this, we performed a controlled experiment in a phytotrone where a number of phenotypic measurements were assessed. Since different organs show very different responses to drought [32–34] we estimated the effects of drought separately in mature leaves and root tips. We performed massive parallel sequencing of RNA (RNA-seq) on the Illumina platform, assembled the sequencing reads *de novo* into gene models and then mapped the reads back to the gene models in order to quantify the level of gene expression per gene model among the different samples. We then performed GO enrichment analyses on the set of differentially expressed genes to identify important functional categories involved in the response to drought in the two tissues and genotypes.

## Results

### Phenotypic responses to drought

**Fig. 1:**
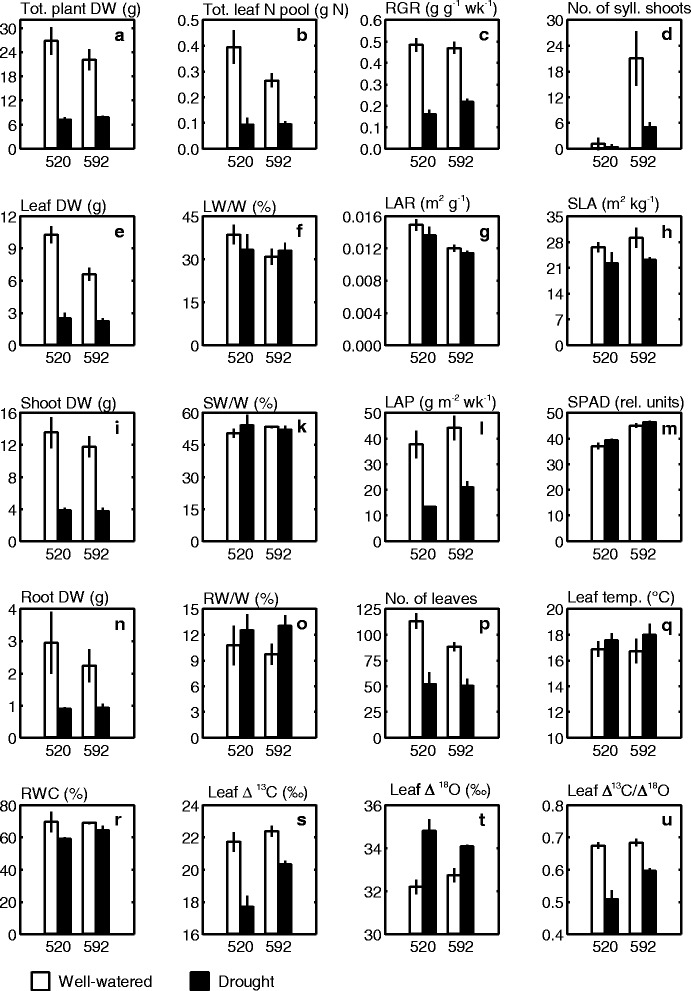
Means (± SD) for various eco-physiological traits in genotype 592 and and 520. The genotypes were grown in a growth chamber (three blocks) and exposed to two experimental conditions (well-watered (white) and drought (black)). DW = dry weight (biomass), RGR = relative growth rate, LAR = leaf area ratio, LAP = leaf area productivity, SLA = specific leaf area, RWC = relative water content, SPAD values indicate leaf chlorophyll content

**Fig. 2:**
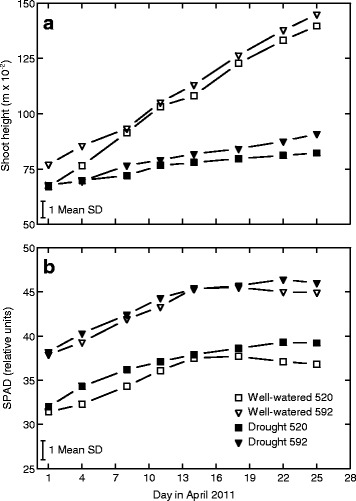
Means for shoot height (**a**) and SPAD value (**b**) in genotype 592 and 520 assessed non-destructively during the experimental treatment period in April. The genotypes were grown in a growth chamber (three blocks) and exposed to two experimental conditions, i.e. well-watered (open symbols) and drought (closed symbols). Mean standard deviation (SD) across all treatments and genotypes is indicated in the lower left corner

Plants grown in WW conditions were both taller (mean (± standard deviation (SD)) 135.5 ± 6.4 cm), had more sylleptic shoots and greater total root, shoot and leaf biomasses compared to the drought stressed plants (height mean 84.3 ± 10.5 cm; Fig. 1a, d, e, i, m; Table 1, main effect treatment). These differences were established already shortly after the onset of the experimental treatments, and the same overall pattern remained across the whole experimental period (Fig. 2a for plant height). Leaf chlorophyll concentration, here assessed in terms of SPAD i.e. leaf nitrogen (N) per leaf area [35], was significantly higher in the drought treatment compared to the WW condition (Table 1), and also that pattern was similar across the entire experimental period (Fig. 2b). In contrast to the leaf chlorophyll (or leaf N) concentration, the total accumulation of N was reduced by drought. Thus, at the end of the experiment, total number of leaves and total N accumulation in leaves, which is a main driver for growth, were greatly reduced by drought (Fig. 1b; Table 1, main effect treatment). The drought-exposed plants also had greater relative biomass allocation to roots compared to the WW plants (Fig. 1n; Table 1, main effect treatment). In contrast to roots, relative shoot and leaf allocation (SW/W, LW/W) was not significantly affected by drought stress (Fig. 1f, k; Table 1).

**Table 1:** ANOVA for various eco-physiological traits measured in genotype 592 and 520 grown in a growth chamber (three blocks) and exposed to two experimental conditions: well-watered or drought

		Source of variation				
Traits		Block	Genotype (G)	Treatment (T)	G x T	Error
		(d.f*.* = 2)	(d.f. = 1)	(d.f. = 1)	(d.f*.* = 1)	(d.f. = 52)
Total plant DW^a^ (g)	MS	0.048	0.055	20.355	0.315	0.059
	*p-*value	*0.452*	*0.338*	**<0.001**	**0.025**	
Total leaf N pool (g)	MS	*0.020*	*0.024*	*0.972*	*0.034*	0.005
	*p-*value	**0.030**	**0.038**	**<0.001**	**0.014**	
RGR^a^ (g g^−1^ wk^−1^)	MS	0.003	0.006	1.188	0.018	0.003
	*p-*value	*0.456*	*0.184*	**<0.001**	**0.025**	
Number of syll. shoots	MS	*50*	*2269*	*1050*	874	46
	*p-*value	0.342	**<0.001**	**<0.001**	**<0.001**	
Leaf DW^a^ (g)	MS	0.343	0.857	31.523	0.057	0.302
	*p-*value	*0.328*	*0.098*	**<0.001**	*0.665*	
LW/W (%)	MS	108	2640	399	1465	220
	*p-*value	*0.614*	**<0.001**	*0.183*	**0.013**	
LAR^a^ (m^2^ g^−1^)	MS	2.87	97.30	13.57	1.39	0.92
	*p-*value	*0.052*	**<0.001**	**<0.001**	*0.223*	
SLA^a^ (m^2^ kg^−1^)	MS	58.0	42.2	390.8	10.7	12.7
	*p-*value	**0.015**	*0.074*	**<0.001**	*0.363*	
Shoot DW^a^ (g)	MS	0.018	0.077	22.310	0.042	0.067
	*p-*value	*0.768*	*0.289*	**<0.001**	*0.434*	
SW/W (%)	MS	441	1410	15	2190	223
	*p-*value	*0.149*	**0.015**	*0.787*	**0.003**	
LAP^a^ (g m^−2^ wk^−1^)	MS	68	728	8080	5	40
	*p-*value	*0.191*	**<0.001**	**<0.001**	*0.729*	
SPAD^a^ (rel. units)	MS	3.6	844.1	46.9	2.3	8.1
	*p-*value	*0.645*	**<0.001**	**0.020**	*0.596*	
Root DW^a^ (g)	MS	0.476	0.321	15.384	0.405	0.126
	*p-*value	**0.029**	*0.116*	**≤0.001**	*0.079*	
RW/W (%)	MS	2490	31	4997	643	114
	*p-*value	**<0.001**	*0.606*	**<0.001**	**0.021**	
No. of leaves	MS	*546*	*2640*	*36803*	1995	231
	*p-*value	0.103	**0.001**	**<0.001**	**0.005**	
Leaf temperature (°C)	MS	9.56	0.19	13.56	1.26	0.70
	*p-*value	**<0.001**	*0.607*	**<0.001**	*0.185*	
RWC^a^ (%)	MS	105.1	86.7	818.2	132.0	199.3
	*p-*value	*0.595*	*0.513*	**0.048**	*0.420*	
Leaf Δ^13^C (‰)	MS	1.7	40.6	138.8	14.5	1.1
	*p-*value	*0.224*	**<0.001**	**<0.001**	**0.001**	
Leaf Δ^18^O (‰)	MS	0.16	0.12	59.12	5.94	0.27
	*p-*value	*0.555*	*0.503*	**<0.001**	**<0.001**	
Leaf Δ^13^C/Δ^18^O	MS	0.002	0.035	0.242	0.023	0.001
	*p-*value	*0.190*	**<0.001**	**<0.001**	**<0.001**	

The ANOVA model included the main effects Block, Genotype and Treatment and the Genotype x Treatment interaction; Block was treated as random factor, and Genotype and Treatment were treated as fixed factors. MS = mean square. d.f. = degrees of freedom

^a^Trait abbreviations: *DW* dry weight (biomass), *RGR* relative growth rate, *LAR* leaf area ratio, *LAP* leaf area productivity, *SLA* specific leaf area, SPAD values indicate leaf chlorophyll content (here assessed two days before the final harvest), *RWC* relative water content P-values ≤ 0.05 are shown in bold

Many traits differed between the two genotypes (Table 1, main effect genotype). At the end of the experiment, mean height growth was significantly higher for genotype 592 compared to 520, both in the WW and in the drought conditions (Fig. 2a; ANOVA: main effect genotype, *p*-value (*p*) = 0.007, not shown). Genotype 592 also produced many more sylleptic shoots than genotype 520, but had fewer leaves and lower total leaf N pool (Fig. 1b, d, o; Table 1, main effect genotype). Mean total biomass at the start of the experimental treatments was similar in the two treatments (ANOVA: main effect genotype *p* = 0.231). The biomass allocation to roots (RW/W) was similar between the genotypes, whilst leaf and shoot allocation varied significantly between the genotypes (Fig. 1f, j, n; Table 1 main effects genotype). There were also several significant interaction effects between genotype and treatment, indicating differential drought responses for the two genotypes. For example, genotype 592 displayed greater drought-induced increase in root biomass allocation (RW/W) than 520; leaf biomass allocation decreased (i.e. 520) and increased (i.e. 592) in response to drought; and shoot biomass allocation increased in response to drought only in genotype 520 (Fig. 1f, j, n; Table 1 Genotype x Treatment interaction (G x T) effects). In contrast, genotype 520 was characterised by stronger drought-induced reduction of leaf number and total leaf N pool, whilst 592 showed stronger drought-induced reduction in the number of sylleptic shoots (Fig. 1b, d, o; Table 1, G x T effects).

Relative growth rate (RGR) was greatly reduced by drought condition (Table 1, main effect treatment), but similar between the genotypes (main effect genotype). The drought-induced reduction in RGR was accomplished by corresponding reductions in leaf area ratio (LAR), specific leaf area (SLA) and biomass productivity per unit leaf area (LAP) (Eqn. 1 & 2) (Table 1, main effect treatment). As indicated by significant genotype by treatment interactions, the reduction of RGR, total plant DW and total leaf N pool by drought was more pronounced in 520 than 592 (Fig. 1a, b, c; Table 1, G x T effects). That genotype (i.e. 520) was characterized by generally higher leaf area ratio (LAR) and lower leaf area productivity (LAP) compared to 592 (eqn 1; Fig. 1g, k; Table 1, main effect genotype). Following eqn. 2, the greater LAR of 520 was mostly a consequence of greater leaf biomass allocation (LW/W; Table 1, main effect genotype; Fig. 1f). In contrast, the higher LAP of 592 was a consequence of greater leaf chlorophyll content (SPAD index; Fig. 1l; Table 1, main effect genotype) in that genotype. Ultimately, the RGR of the high-SPAD genotype (i.e. 592), was less affected by drought compared to the high-LAR genotype (i.e. 520).

Leaf temperature and relative water content (RWC) varied between growth conditions, but were similar between genotypes (Fig. 1p, q; Table 1, main effect treatment and genotype). In drought condition, RWC decreased, leaf temperature increased, stomata were more closed (higher leaf Δ^18^O indicates lower stomatal conductance, *g*_*s*_), intrinsic water use efficiency was higher (lower leaf Δ^13^C indicates higher intrinsic water use efficiency), and also carboxylation efficiency was higher (lower ratio of Δ^13^C and Δ^18^O indicates higher carboxylation efficiency) (Fig. 1p-t, Table 1, main effect treatment). Genotype affected isotope ratios, and the genotype 592 had lower intrinsic water use efficiency (i.e. higher leaf Δ^13^C) and lower carboxylation efficiency (i.e. higher ratio of Δ^13^C and Δ^18^O) than genotype 520 (Table 1, main effect genotype). In general, leaf gas exchange as reflected by the isotope data indicates small genotype differences under WW conditions along with great genotype differences in drought conditions. This pattern was statistically supported by highly significant interaction effects in all isotope measures (Fig. 1r-t, Table 1). More specifically, the drought-induced decrease in stomatal conductance (i.e. increase in leaf Δ^18^O) was less pronounced in 592 compared to 520. Also drought-induced increase in intrinsic water use efficiency (i.e. decrease in leaf Δ^13^C) was less pronounced in 592 than in 520 (Table 1, G x T effects).

### *De novo* assembly and identification of DEGs

Illumina sequencing of 36 libraries prepared from RNA extracted from leaf and root tip tissues from two willow genotypes generated 7.91 × 10^8^ paired-end sequencing read pairs that corresponds to 158 × 10^9^ base pairs (bp) (Table 2). Sequencing read pair numbers per library varied between 18.1 and 33.3 million and did not differ significantly between tissues (ANOVA: *p* = 0.60), genotypes (ANOVA: *p* = 0.55) or treatment (ANOVA: *p* = 0.39). When all sequencing reads were combined and assembled *de novo* using the Trinity assembler, 91,701 contigs were obtained, which represented 52,599 gene models. After filtering on gene expression(fragments per kilobase of transcript per million mapped fragments (FPKM) ≥ 1), 35,733 gene models remained of which 24,421 contained open reading frames (Additional file 1). The gene models in this high confidence gene set ranged in size from 301 to 16,559 bp with a mean length of 1485 bp and a median length of 1273 bp (Additional file 2). 40.6 % of all reads mapped to the high confidence gene set with the parameters used for expression analysis. The majority of them (99.7 %) mapped to one unique position. More detailed mapping statistics can be found in Table 2. The program *edgeR* was used with the “calcNormFactors()” function to identify DEGs between drought and WW conditions in: 1) leaves of genotype 592 (five replicates), 2) leaves of genotype 520 (five replicates), 3) root tips of genotype 592 (four replicates) and 4) root tips of genotype 520 (four replicates). Normalized read counts are listed in Additional file 3 and normalization factors used in the *edgeR* analyses are listed in Additional file 4. Genes were defined as upregulated DEGs if false discovery rate (FDR) was ≤ 0.05 and log_2_ fold change (FC) ≥ 1 and as downregulated DEGs if FDR was ≤ 0.05 and log_2_ FC ≤ −1. A total of 6935 DEGs were identified across genotypes and tissues, representing 5112 unique gene models. 3082 DEGs were upregulated (of which 2175 were unique) and 3853 were downregulated (of which 2974 were unique) (Table 3, Additional file 5). Much fewer DEGs were found in the leaves than in the root tips (Table 3, Additional file 5), a pattern that was consistent across both genotypes. This result manifests the small effects of drought on the overall transcription in the young mature leaves in these two genotypes and shows that root tips instead have a much stronger transcriptional response to drought than leaves do. When comparing the two genotypes, more DEGs were found in the leaves of genotype 520 compared to genotype 592, while the number of DEGs in the root tips was more similar between the genotypes (this was particularly evident for the upregulated DEGs) (Table 3). Twenty-eight genes (Additional file 5) displayed significant G x T effects when using a generalized linear model and the gene ontology (GO) term ADP-binding was significantly enriched among these genes. When comparing the drought responses in the leaves and the root tips of the two genotypes for these genes, 25 displayed greater drought responses in leaves than in the root tips. The hypothesis that there was an equal chance that the effect is stronger in either tissue could thus be rejected (*p* = 2.74 × 10^−5^, binomial test see Additional file 6). This result further strengthens the finding of greater genotypic differences in the drought responses in the leaves than in the roots.

**Table 2:** Summary of Illumina sequencing and mapping of the sequencing read to the genes in the high confidence gene set

	Total reads	Total mapped reads	% mapped reads^a^	# uniquely mapped reads	% uniquely mapped reads^b^
520 leaves WW	208,238,566	85,078,408	40.86	84,848,522	99.73
520 roots WW	177,073,828	70,448,906	39.79	70,245,780	99.71
520 leaves drought	223,031,526	111,012,324	49.77	110,732,434	99.75
520 roots drought	170,916,478	70,687,714	41.36	70,463,550	99.68
592 leaves WW	202,996,992	96,790,050	47.68	96,548,610	99.75
592 roots WW	185,878,236	90,739,420	48.82	90,492,450	99.73
592 leaves drought	234,211,410	63,601,116	27.16	63,220,510	99.40
592 roots drought	178,929,458	52,870,112	29.55	52,564,102	99.42
Total	1,581,276,494	641,228,050	40.55	639,115,958	99.67

^a^Relative to the total number of reads

^b^Relative to the number of mapped reads

**Table 3:** Number of DEGs between drought and well-watered conditions for each genotype and each tissue

Genotype	592	520	592	520	
Tissue	Leaves	Leaves	Root tips	Root tips	Total no. of DEGs
Upregulated DEGs FDR ≤ 0.05, log_2_ FC ≥ 1	1	126	1457	1498	3082
Downregulated DEGs FDR ≤ 0.05, log_2_ FC ≤ −1	34	123	2163	1533	3853
Total no. of DEGs	35	249	3620	3031	6935

### Functional annotation and GO enrichment analysis

The Blast2GO software tool was used to functionally annotate the *de novo* assembled gene products and for GO enrichment analyses in order to identify functions and genes involved in drought stress responses in the leaves and root tips. Of the 24,421 genes in the high confidence gene set, 15,980 were annotated with a GO term.

The GO enrichment analysis was done for up and downregulated annotated DEGs in leaves and root tips for each genotype separately using the annotated high confidence gene set as reference. All enriched GO terms in every comparison are presented in Additional file 7. Overall, few GO terms were enriched for the DEGs in the leaves, in fact none was significantly enriched for the DEGs in genotype 592, suggesting that drought had little impact on the function of the leaves in this genotype. However, genotype 520 showed a greater functional drought response as several GO terms were significantly enriched, particularly for the upregulated DEGs. For example at the molecular function (F) ontology level, “peptidyl-prolyl cis-trans isomerase activity” was one of the most significantly overrepresented terms and at the biological process (P) ontology level, different response and regulation terms were significantly overrepresented (Additional file 7). At the cellular component (C) ontology level, several terms related to the thylakoid and the cell wall were overrepresented (Additional file 7). For the downregulated DEGs at the F ontology level, “oxygen binding” was the most significantly overrepresented term (Additional file 7). At the P ontology level, “arachidonic acid metabolic process” and “epoxygenase P450 pathway” were overrepresented. No term was significantly overrepresented at the C ontology level.

**Fig. 3:**
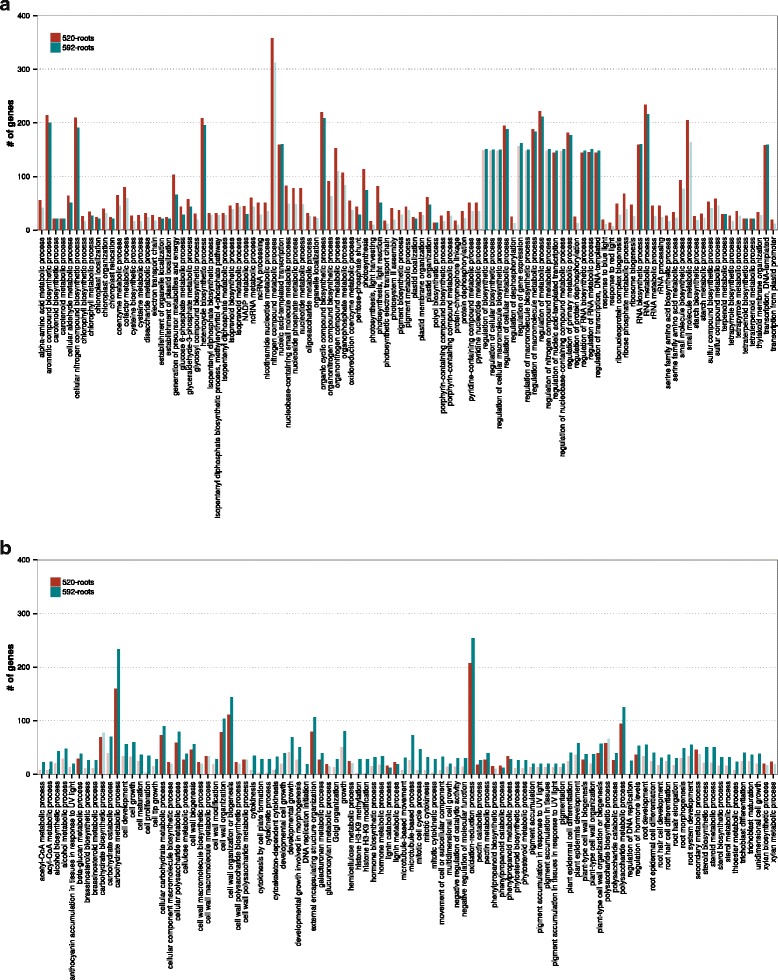
Over-represented GO terms at the *biological process* ontology level for up- (**a**) and downregulated (**b**) DEGs in the root tips of genotype 592 and genotype 520. The red bars show the number of DEGs annotated with the GO terms in the root tips of genotype 592 and the blue bars show the number of DEGs annotated with the GO terms in the root tips of genotype 520. GO terms are presented if they were enriched (FDR < 1 × 10^−4^) in at least one of the two genotypes and if at least ten genes were enriched for that term. Solid bars represent a significant over-representation of the GO term in this genotype while fainted bars are given as reference if the significance level was not reached

Many more GO terms were enriched for the DEGs in the root tips compared to the leaves, which demonstrates the greater drought responses in this tissue compared to the leaves. In Fig. 3 we present overrepresented GO terms (FDR < 0.0001) in the P ontology level associated with upregulated (Fig. 3a) and downregulated (Fig. 3b) DEGs for both genotypes. Many GO terms were significantly or nearly significantly enriched in both genotypes, which means that the genotypes displayed overall similar responses to drought in the root tips. For the upregulated DEGs, the most affected functions were associated with biosynthetic and metabolic processes (Fig. 3a).

### A core set of candidate genes involved in the drought response

To identify candidate genes involved in drought stress responses in the two willow genotypes, we extracted the upregulated DEGs in the root tips of both genotypes annotated with any of the 15 “response” GO terms listed in Table 4. This resulted in a list with 115 genes for genotype 592 and 141 genes for genotype 520 (Additional file 8). Based on annotations and reported functions in stress responses, a core set of 28 candidate genes were identified (Table 5) that could function as targets for detailed functional studies of drought responses at the molecular level in willows and related species. Several of the genes were homologous to genes encoding transcription factors, e.g. MYBs, WRKYs, bZIPs and heat stress transcription factors with known functions in stress responses. Other genes were homologous to genes encoding dehydrin and chlorophyll a/b binding proteins.

**Table 4:** GO terms associated with upregulated DEGs in the root tips that were enriched (FDR ≤ 0.05) in at least one of the genotypes and used to select candidate genes with putative functions in drought responses in willows

GO-ID	Term	FDR	No of genes in test set	No of genes in ref set
		Genotype 592/520	Genotype 592/520	Genotype 592/520
GO:0009628	Response to abiotic stimulus	7.9 × 10^−3^/1.8 × 10^−3^	106/114	1004/996
GO:0009644	Response to high light intensity	2.4 × 10^−2^/2.6 × 10^−2^	15/15	73/73
GO:0009642	Response to light intensity	4.9 × 10^−3^/5.7 × 10^−3^	19/19	90/90
GO:0009636	Response to toxic substance	3.5 × 10^−2^/-	5/-	8/-
GO:0009415	Response to water	4.9 × 10^−2^/-	17/-	96/-
GO:0042538	Hyperosmotic salinity response	-/3.2 × 10^−2^	-/9	-/31
GO:0098542	Defense response to other organism	-/9.3 × 10^−3^	-/47	-/342
GO:0009637	Response to blue light	-/1.8 × 10^−5^	-/19	-/51
GO:0010218	Response to far red light	-/1.1 × 10^−4^	-/15	-/37
GO:0009416	Response to light stimulus	-/1.4 × 10^−2^	-/55	-/432
GO:0009314	Response to radiation	-/2.6 × 10^−2^	-/57	-/466
GO:0010114	Response to red light	-/5.1 × 10^−5^	-/14	-/29
GO:0051788	Response to misfolded protein	-/3.4 × 10^−2^	-/0	-/93
GO:0002679	Respiratory burst involved in defense response	2.5 × 10^−2^/-	7/-	19/-
GO:0006974	Cellular response to DNA damage stimulus	4.9 × 10^−2^/1.9 × 10^−2^	6/6	256/256

**Table 5:** A core set of candidate genes with reported or putative functions in stress responses

Sequence ID	Description	BLAST E-value^a^	Protein description at TAIR obtained by BLASTX with the *Salix* sequence
c34790_g1_i6	ap2-like ethylene-responsive transcription factor ail5	5.2 × 10^−75^	Encodes a member of the AP2 family of transcriptional regulators.
c19050_g1_i3	bzip transcription factor 60	4.2 × 10^−144^	Consists of a bZIP DNA binding domain followed by a putative transmembrane domain.
c25859_g1_i2	Chlorophyll a-b binding protein chloroplastic	6.5 × 10^−161^	Photosystem II encoding the light-harvesting chlorophyll a/b binding protein CP26 of the antenna system of the photosynthetic apparatus.
c16933_g1_i1	Chlorophyll a-b binding chloroplastic	2.5 × 10^−175^	PSI type II chlorophyll a/b-binding protein (Lhca2*1) mRNA.
c3652_g1_i1	Chlorophyll a-b binding protein chloroplastic	5.1 × 10^−157^	Encodes a component of the light harvesting complex associated with photosystem I.
c28579_g1_i2	Chloroplast stem-loop binding protein of 41 kda chloroplastic	0	Encodes CHLOROPLAST RNA BINDING (CRB), a putative RNA-binding protein.
c22127_g1_i1	Dehydrin	5.0 × 10^−93^	Encodes a gene induced by low temperature and dehydration.
c16392_g1_i1	Ethylene-responsive transcription factor 4-like	4.1 × 10^−42^	Encodes a member of the ERF (ethylene response factor) subfamily B-1 of ERF/AP2 transcription factor family (ATERF-4).
c20465_g2_i1	Heat shock transcription factor a2 isoform 1	1.3 × 10^−57^	Member of Heat Stress Transcription Factor (Hsf) family.
c23076_g1_i1	Heat stress transcription factor a-3	0	Member of Heat Stress Transcription Factor (Hsf) family. Expression is regulated by DREB2A.
c33073_g3_i1	Heat stress transcription factor a-6b	2.9 × 10^−98^	Member of Heat Stress Transcription Factor (Hsf) family.
c14799_g1_i1	Heat stress transcription factor a-6b-like	5.7 × 10^−127^	Member of Heat Stress Transcription Factor (Hsf) family
c32554_g10_i1	Heat stress transcription factor b-2b	5.1 × 10^−96^	Member of Heat Stress Transcription Factor (Hsf) family
c29038_g2_i1	Heat stress transcription factor c-1	5.8 × 10^−166^	Member of Heat Stress Transcription Factor (Hsf) family
c31828_g1_i3	Mitochondrial transcription termination factor family protein isoform 1	0	Encodes a putative mitochondrial transcription termination factor.
c6068_g1_i1	myb-like transcription factor partial	1.2 × 10^−101^	Putative transcription factor MYB108 (MYB108) mRNA.
c21976_g1_i1	myb transcription factor family protein	2.9 × 10^−179^	Encodes a MYB transcription factor involved in wounding and osmotic stress response. Member of the R2R3 factor gene family.
c35418_g2_i1	Photosystem ii 10 kda chloroplastic	8.9 × 10^−75^	Encodes for the 10 kDa PsbR subunit of photosystem II (PSII).
c26360_g1_i3	Photosystem ii stability assembly factor chloroplastic	0	Encodes a stability and/or assembly factor of photosystem II.
c34906_g2_i3	Probable wrky transcription factor 33	0	Member of the plant WRKY transcription factor family. Involved in response to various abiotic stresses.
c31974_g2_i2	Probable wrky transcription factor 40	1.7 × 10^−178^	Pathogen-induced transcription factor.
c26874_g1_i3	Probable wrky transcription factor 48	4.9 × 10^−151^	Encodes WRKY48, a member of the WRKY Transcription Factor. WRKY48 is a stress- and pathogen-induced transcriptional activator that represses plant basal defense.
c27657_g4_i1	Protein odorant1-like	0	Encodes a MYB transcription factor involved in wounding and osmotic stress response. Member of the R2R3 factor gene family.
c3736_g1_i1	Protochlorophyllide chloroplastic	1.1 × 10^−82^	Encodes for a protein with protochlorophyllide oxidoreductase activity. The enzyme is NADPH- and light-dependent.
c24362_g1_i1	Protochlorophyllide reductase-like	0	Encodes for a protein with protochlorophyllide oxidoreductase activity. The enzyme is NADPH- and light-dependent.
c21511_g1_i2	Stress enhanced protein chloroplastic	1.7 × 10^−80^	Encodes a stress enhanced protein that localizes to the thylakoid membrane and whose mRNA is upregulated in response to high light intensity. It may be involved in chlorophyll binding.
c34938_g1_i3	BZIP17, transcription factor	0	bZIP17 appears to regulate transcription as part of a salt and osmotic stress response.
c21535_g1_i1	Transcription factor myb108-like	7.1 × 10^−150^	Member of the R2R3 factor gene family.

^a^E-value between the sequence and the best hit in the Viridiplantae subset of the NCBI nr database

## Discussion

### Genotype specific physiological responses to drought

This study accommodated a growth experiment in which cuttings of two genotypes were grown in an irrigation contrast for sufficiently long time to develop considerable variation both at the transcriptional and phenotypic levels. We demonstrated that the two genotypes displayed contrasting phenotypic responses to drought. Genotype 592 was overall less affected by drought as it displayed a weaker growth reduction. The weaker growth reduction of 592 was associated with greater increase in root biomass allocation (RW/W) in response to drought, enhancing the capacity to explore available water and nutrient resources. Interestingly, genotype 592 also displayed generally higher foliar SPAD values, which is in agreement with the hypothesis that higher area-based leaf N content is an acclimation to drought [36]. In contrast to 592, genotype 520 displayed generally higher evapotranspiration area (e.g. LAR) and responded to drought with strong leaf area reduction, greatly decreased stomatal conductance and increased intrinsic water use efficiency. Thus, in addition to the observed genotypic variation in mean traits (e.g. LAR, LAP, SPAD), we also found indications for genotype differences in trait variability in a drought contrast, especially for the stomatal physiology traits (leaf Δ^13^C and Δ^18^O). Genotypic differences in trait variability, observed in a drought contrast, may be indicative of genotypic variation in trait plasticity in relation to drought. Large genotypic variation in trait plasticity of leaf traits similar to the ones in our study was found among *Eucalyptus* provenances originating from a rainfall gradient in Australia, and interpreted as important features in the climate adaptation of these trees [37].

### Generation of the high confidence gene set

*De novo* assembly of all sequencing reads generated 52,599 gene models and after filtering out lowly expressed gene models, 35,733 remained. In a next filtering step, gene models that did not contain an open reading frame were filtered out, leaving 24,421 gene models in the high confidence gene set. Out of these, 18,128 were functionally annotated with a GO term in Blast2Go. Although it is problematic to compare these numbers with those from other studies as different tissues and also filtering criteria are often used, *de novo* assembly of RNA-seq data in *P. pruinosa* resulted in 48,653 gene models [38] and in *S. matsudana* 48,817 unigenes were retrieved [39], showing that the figures were overall rather similar across the different species. The number of annotated genes in this high confidence set was considerably higher than the number of gene models annotated with a GO term in *P. pruinosa* and *P. euphratica* where 11,587 [40] and 9296 in [9] were reported respectively, possibly reflecting improved annotations tools.

### Variation in the number of DEGs and enriched GO terms between the two genotypes and tissues

When comparing the responses to drought between the two tissues we found that drought affected gene expression of many more genes in the root tips compared to the leaves, a result that was consistent across both genotypes. A similar pattern was previously observed in hybrid poplars (*P. deltoides* × *P. nigra*) [7] where about twice as many probe sets displayed a significant change in response to drought in the root tips compared to the mature leaves. The authors proposed that this difference could in part be explained by the higher sensitivity to water deprivation in an actively growing tissue, i.e. the root tips. Judging from the number of DEGs in the root tips, the two genotypes displayed rather similar responses. This similarity was also evident in the enriched GO terms associated with these DEGs that were present in both genotypes. A somehow more unexpected result was the numerous enriched terms in the cellular component level that was related to the chloroplast. The overall lack of response of drought on gene expression in the mature leaves was also unexpected as several previous studies have reported a greater response in leaves. For instance, in *P. trichocarpa* 5689 DEGs were identified in leaves [10], in *P. euphratica*, where 5083 genes were differentially expressed in leaves [8] and in hybrid poplar (*P. deltoides* × *P. nigra*) 2120 were affected in leaves [7]. Results from the present study instead resemble the results in *P. balsamifera* where 98 probe sets displayed increased transcript abundance to drought [11]. Less responses were also seen in shoots of *P. balsamifera* [6]. In contrast to the root tips, the genotypes displayed a greater difference in the responses in the leaves with much fewer DEGs and enriched GO terms in genotype 592 than in genotype 520. Strong genotypic effects were previously found in leaves of hybrid poplars [7] and *P. balsamifera* (six genotypes) [11]. This was also seen in shoots of hybrid poplar clones (*P. deltoides* × *P. nigra* and *P. nigra* × *P. maximowiczii*) [6]. Interestingly, genotype 592 also displayed less physiological responses at the leaf level compared to genotype 520 supporting a correlation between transcriptional and physiological responses. For example, the drought-induced shifts in stomatal physiology (as reflected by Δ^13^C and Δ^18^O) were more pronounced in the genotype 520, which coincides with the overrepresented GO terms “thylakoid” and cell wall in the cellular component ontology level. Among the 28 genes that displayed significant G x T effects, several encoded disease resistant proteins and leucine-rich repeat proteins. An exciting hypothesis is that the two genotypes carry different alleles at these genes rendering different drought responses.

### A core set of candidate genes involved in drought stress responses

We have produced a core set of candidate genes that we find particularly interesting and that should be further examined for their role in the molecular stress responses and we discuss some of them here. Among our candidate genes were several transcription factors i.e. MYBs, bZIPs and WRKYs, with known functions in ABA-dependent stress responses [41]. They among other things regulate stress responses via modulation of transcription of downstream genes. Furthermore, several candidate genes encoded chlorophyll *a*/*b* binding proteins (i.e. CABs). Typical for this protein family is the CAB domain, containing the amino acid residues involved in pigment binding. There are some indications that they function in high-light protection in a broad sense and that they encode homologs to light-harvesting-like proteins (LILs). All organisms performing oxygenic photosynthesis also contain LILs [42], which are highly homologous to the higher plant light-harvesting antenna and contain the CAB domain. The LILs are regulated opposite to the light-harvesting complex proteins (LHCs); under high light condition - when the expression of LHCs is repressed - the LILs are upregulated. While in cyanobacteria LILs have been found to respond to different stresses, their function in plant was thought to be restricted to light stress [43]. It is thus possible that these genes and gene products play an important role in the stress response – even in non-photosynthetic tissue. Also in Arabidopsis [44] and rice [45] enhanced expression of such genes was observed in roots exposed to drought stress, indicating a general importance of these genes independent of the species.

## Conclusions

In this study we report the first large transcriptome study in willows where we have compared physiological and transcriptional responses to drought between two genotypes and two tissues. A *de novo* assembly and subsequent filtering of root tip and leaf transcriptomes across the two genotypes and two conditions, generated a set with 24,421 genes. A total of 5112 unique DEGs were identified between the drought and WW conditions, of which the majority were found in the root tips. We also found substantial differences in drought responses between the genotypes, both at the phenotypic and trancriptional level. Phenotypically, genotype 592 displayed less growth reduction in response to drought compared to genotype 520, which suggests that this genotype is more drought adapted than genotype 520. In addition to the genotypic variation in several mean traits, we also found considerable genotypic variation in trait plasticity (especially leaf traits), which may play a role in drought adaptation. At the transcriptional level, genotype 520 displayed a greater response in the leaves as many more DEGs were found compared to genotype 592. As the two genotypes displayed overall similar transcriptional responses in the root tips, but more variation in the leaves, it is possible that the observed phenotypic differences between the genotypes are due to transcriptional differences in their leaves. We also identified a core set of candidate genes encoding proteins with a putative function in drought response, for example MYBs, bZIPs and WRKYs and chlorophyll a/b binding proteins. Knowledge from this study increases our understanding of the physiological and molecular basis of drought responses in willows, however these results are applicable to all woody plants.

## Methods

### Plant material

Two *Salix* genotypes (520 and 592) from the S_1_ pedigree (*N* = 463) [31] were used in this study. S_1_ is a cross between the female clone 78183, a diploid *S. viminalis* and Björn, a diploid *Salix viminalis* L × *S. schwerinii* E Wolf hybrid male. Results from previous greenhouse and phytotron experiments showed that both genotype 520 and 592 were high producing clones, both in WW and in drought conditions with repeated periods of drought [30]. Interestingly, they displayed different responses to drought, as an example, genotype 592 lost many more leaves than 520 [30].

The S_1_ population is planted and conserved in a plant archive owned by the Swedish University of Agricultural Sciences, near Uppsala (59°49’ N 17°40’ E, central Sweden) where dormant shoots from the two genotypes were collected the 2^nd^ of February 2011 and stored in −4 °C. On the 17^th^ of February, fifty, six cm long cuttings were prepared for each genotype and planted in 3-L pots filled with commercial soil (“Weibull’s Krukväxtjord Lera & Kisel”, organic matter 95 %; pH 5.5-6.5; 182 g/m^3^ N, 91 g/m^3^ P, 195 g/m^3^ K, maximum 50 g/m^3^ micro nutrients). The relatively small pot size and the resulting high biomass to substrate volume ratio at final harvest has probably decreased growth in all genotypes and treatments compared to field conditions [46], but visible observation revealed that roots never occupied more than approximately half of the substrate volume per pot at final harvest. The experiment was conducted in one walk-in growth chamber where the plants were organized in three groups. Each group consisted of equal number of plants per genotype, thus each group represented one block or replicate. The plants were grown for 38 days under 20 °C constant temperature, 70 % relative humidity and 16 h photoperiod (300 μmol PAR m^−2^ s^−1^) with regular watering. The positions of the plants within the blocks as well as the positions of the blocks in the chamber were changed every day and the plants were pruned so that only the main shoots were preserved. For the drought treatments starting the 28^th^ of March, 30 plants at the same growth stage of each genotype were selected, of which 15 were assigned as controls with regular watering (well-watered or WW condition) and 15 subjected to drought stress (drought condition) for 31 days. The aim was to keep the drought stressed plants just above their wilting point throughout the treatment period. The wilting point or the minimal amount of soil moisture the plants required not to wilt was therefore studied in six plants for each genotype prior to the drought stress treatment was initiated. The plants were weighed daily until they started to wilt (no water was supplied). To apply drought stress, the onset of visible wilting signs (“wilting point”) of individual plants was used as a physiological drought stress indicator. Thus, in the drought treatment, any water supply to individual pots was initially withheld until the plants had reached their individual wilting points, at which pot weights were determined. At a daily basis throughout the experimental period in April, all pots were weighed and water was added in the necessary amounts to keep the substrate at a more or less constant minimum water supply level. Applying this procedure, all plants in the drought stress condition were kept close to their wilting points throughout the whole treatment period. The WW plants were regularly watered to field capacity of the soil throughout the treatment period and weighed every day. To account for increased consumption as the plants grew, watering quantities were slightly increased throughout the treatment period. When the drought stressed plants were kept near the wilting point, they received approximately 50 % of the water supplied to the WW plants.

### Eco-physiological measurements

Two harvests were carried out during the experiment, one initial harvest just before the start of the treatment period (28^th^ of March) and a final harvest at the end of the treatment period (27^th^ - 28^th^ of April). The harvested plants (five plants per block, treatment and genotype) were separated into leaves, stems, the original cutting, roots, sylleptic shoots and sylleptic leaves and all parts were oven dried for 48 h at 70 °C. Excised leaves were not included. Leaf areas were determined on fresh leaves with an area meter (LI-3100, LiCor Inc., Lincoln, NE, USA). Both for the initial and the final harvest, the dry weights of each plant compartment as well as leaf areas of all plants were assessed. The total dry weight (DW) per plant was estimated as the sum of all plant compartments excluding the cutting. Height was assessed non-destructively throughout the treatment period. Height was defined as length from the base of the shoot to the thickest part of the apical bud. Leaf chlorophyll content (SPAD index) of one leaf (per plant) in the upper middle canopy was measured non-destructively at the same occasions as plant height by using a portable chlorophyll meter (SPAD-502, Konica Minolta Sensing Inc. Japan). In *Salix* spp. the SPAD index has previously been shown to be closely related to leaf nitrogen (N) content [35]. Also leaf temperature was measured on the same leaf using a portable infrared thermometer (IR400, Extech Instruments). An average of five measurements per leaf was used for both assessments. In general, relative water content (RWC) is a measure of drought resistance [47] and was measured on leaves sampled from all plants at the day of the final harvest. For determination of RWC, two fully developed leaves per plant were sampled. A simplified measure of RWC was determined as described by Weih and Nordh [48] and indicates here the rate of water loss from the leaf tissue. Leaves were weighted directly after sampling (wt0) and placed on sheets of filter paper in room temperature. Leaves were weighted again after 4 h (wt4) and after they had oven dried for 24 h at 70 °C (wt24). RWC was calculated according to the following formula: RWC (%) = (wt4 – wt24) / (wt0 – wt24) × 100.

The sampled leaves were used for total nitrogen (N); carbon and oxygen isotope analysis performed by Iso-Analytical Limited, Crewe, UK. All analyses were conducted by Elemental Analyser Isotope Ratio Mass Spectrometry (EA-IRMS, Europa Scientific 20-20). Leaf N concentrations were multiplied with total leaf DW of all leaves per plant to obtain total leaf N pool. Leaf carbon and oxygen isotope measurements reflect important characteristics of gas exchange at leaf level and integrate over the lifetime of the leaf. Thus, leaf Δ^13^C is correlated with intercellular CO_2_ concentration *C*_*i*_, and higher *C*_*i*_ indicates lower intrinsic water use efficiency [49]. Leaf Δ^18^O is a measure of stomatal conductance (*g*_*s*_), and increased leaf Δ^18^O is correlated with decreased *g*_*s*_ [49, 50]. Finally, the ratio of Δ^13^C and Δ^18^O (i.e. *C*_*i*_/*g*_*s*_) represents an estimate of carboxylation efficiency, and higher *C*_*i*_/*g*_*s*_ is correlated with lower carboxylation efficiency [51]. The reference material used for δ^13^C analysis of the samples was IA-R001 (wheat flour, δ^13^C_V-PDB_ = −26.43 ‰). Carbon isotope ratio of leaf samples (δ^13^C_sample_) is expressed as: δ^13^C_sample_ (‰) = (R_sample_ / R_PDB_ – 1) × 1000, where R_sample_ and R_PDB_ are the ^13^C/^12^C molar abundance ratio of the leaf material and PeeDee Belemnite standard. Carbon isotope discrimination (Δ^13^C, ‰) was calculated assuming the isotope composition of atmospheric air (δ^13^C_air_) to be −8 ‰ [51] and computed as: Δ^13^C = (δ^13^C_air_ – δ^13^C_sample_) / (1 + δ^13^C_sample_/1000). The reference material used for δ^18^O analysis was IAEA-CH-6 (sucrose, δ^18^O_V-SMOW_ = 36.4 ‰). The δ^18^O of the irrigation water (CO_2_:H_2_O equilibrium technique) was −13.8 ‰. The ^18^O enrichment over the irrigation water (Δ^18^O_sample_) was computed as: Δ^18^O_sample_ (‰) = δ^18^O_sample_ – δ^18^O_irrigation water_. The isotope ratio was calculated as Δ^13^C / Δ^18^O_sample_ [51].

Changes in plant growth over time were analyzed using the methods of classical growth analysis [52] and based on biomass and leaf area changes between the two consecutive harvests. Thus possible differences in relative growth rate (RGR, final plant biomass per initial plant biomass and week, g g^−1^ wk^−1^) between genotypes were related to differences in leaf area ratio (LAR, leaf area per total plant biomass, m^2^ g^−1^); leaf area productivity (LAP, total biomass growth per leaf area and week, g m^−2^ wk^−1^); specific leaf area (SLA, leaf area per leaf biomass, m^2^ kg^−1^); and leaf biomass fraction (LW/W, leaf biomass per total plant biomass, %). The following relationships among growth traits were utilized to analyze differences among the treatments and clones [53]:

1 $$ \mathrm{R}\mathrm{G}\mathrm{R}=\mathrm{LAR}\times \mathrm{LAP} $$

2 $$ \mathrm{LAR}=\mathrm{S}\mathrm{L}\mathrm{A}\times LW/\mathrm{W} $$

### RNA extractions and sequencing

The day before the final harvest, two fully developed young leaves and about one centimetre of several root tips were collected from each plant that were immediately after harvesting snap frozen in liquid nitrogen and stored in −80 °C awaiting RNA extractions. Total RNA was extracted from 40 plants representing five biological replicates from leaves and root tips from the two genotypes in drought and WW conditions. Approximately 100 mg of leaves and 30 mg of root tips were taken for RNA extractions using Spectrum Plant Total RNA Kit (Sigma-Aldrich) with the On-Column DNase I digestion set (Sigma-Aldrich). After quality checking on a 2100 Bioanalyzer (Agilent), four root tip samples were discarded, leaving a total of 36 RNA samples for sequencing. One sequencing library was prepared for each of the 36 samples. The RNA samples were first treated with DNase, then one library per sample was prepared using Illumina’s TruSeq RNA Sample Prep Kit v1, where polyA-fragments were selected, followed by cDNA synthesis and ligation of amplification and sequencing adapters. Sequencing libraries were individually barcoded and then pooled with nine libraries per lane on an Illumina HiSeq 2000 instrument. All samples were sequenced as paired-end, and for 27 samples the read lengths were 100 bp and for nine samples the read lengths were 107 bp for read 1 and 144 bp for read 2. Library preparations and sequencing was performed by the SNP&SEQ Technology Platform in Uppsala.

### *De novo* assembly and filtering

Sequencing reads from all 36 libraries were combined and assembled *de novo* using the Trinity software (version 20140717) [54] with the read trimming and digital normalization options. Default parameters were implemented except for maximum coverage of 50 in the normalization step and a minimum k-mer coverage of 20 as well as a minimum contig length of 300 in the assembly step. The contigs generated in Trinity are by default clustered in “trinity components” or gene models, where each gene model represents a putative gene. The assembly was filtered based on gene expression levels where gene models that had no contig with FPKM (fragments (read pairs) per kilobase of transcript per million mapped fragments) above or equal to 1 were filtered out. For every gene model, the contig with the highest FPKM value was retained. FPKM values were obtained by mapping the sequencing reads (from all libraries) to the contigs with the program RNA-Seq by Expectation-Maximization (RSEM) v. 1.2.12 [55]. In a second filtering step, only gene models that contained and open reading frame was retained. TransDecoder version 20140704 [56] was used with default options to predict open reading frames in the gene models from the filtered *de novo* assembly. This assembly is named the high confidence gene set, which was the dataset used for gene expression analyses.

### Differential gene expression analysis

In order to identify genes with a putative function in the drought response, differentially expressed genes between drought and WW conditions were identified. Gene specific read counts for the genes in the high confidence gene set were obtained using the RSEM v. 1.2.12 [55], that internally uses bowtie v. 1.0.0 [57]. All sequencing libraries were mapped separately to this assembly. The applied mapping parameters allowed for up to two mismatches to reflect the allelic variation. Using the read counts obtained by RSEM, the *R/Bioconductor* package *edgeR* [58] was used to detect differentially expressed genes (DEGs) among the genes in the high confidence gene set between the drought and WW condition in leaves of genotype 592, leaves of genotype 520, root tips of genotype 592 and root tips of genotype 520. The four pairwise comparisons were made using a classic Fisher exact test. For all the analyses in *edgeR*, the read counts were normalized for RNA composition using the “calcNormFactors()” function. Genes that showed significant G x T effects were identified using a generalized linear model (GLM) in *edgeR* with an interaction model (Treatment + Tissue + Genotype + (Treatment × Tissue) + (Genotype × Treatment) + (Tissue × Genotype) + (Treatment × Tissue × Genotype)). Genotype × Treatment interaction terms were retained. In order to quantify whether the genotypes differed more in their drought responses (drought vs. WW conditions) in the leaves compared to the root tips, a statistic test was constructed and implemented to the genes with significant G x T effects (the test is described in Additional file 6). For all tests, genes were defined as differentially expressed if false discovery rate (FDR) was ≤ 0.05 and log_2_ fold change (FC) ≥ 1.

### Functional annotation and GO enrichment analysis

Genes in the high confidence gene set were functionally annotated with GO terms in Blast2GO (version 3.1) [59]. The blast step was performed locally against the Viridiplantae subset of the NCBI nr database downloaded 2015-08-06. All up- and downregulated DEGs for leaves, root tips in the two genotypes were tested for GO enrichment using the Fisher's exact test, which uses the Gossip software integrated in the Blast2GO software. The rationale was to test for over- or underrepresentation of GO terms among the DEGs compared to all annotated genes in the high confidence gene set, using a cut-off threshold of FDR ≤ 0.05. The analyses were performed for the three ontology levels, biological processes (P), molecular function (F) and cellular component (C).

### Availability of supporting data

The raw sequencing reads are available in the European Nucleotide Archive (ENA) with the reference number PRJEB10883 (http://www.ebi.ac.uk/ena/data/view/PRJEB10883).

## Additional files

Additional file 1:
**Sequences of all gene models in the high confidence gene set.** (TXT 40356 kb) Additional file 2:
**Length distribution of transcripts in filtered and high confidence gene set.** (EPS 33 kb) Additional file 3:
**Normalized read counts used in** ***egdeR.*** (XLSX 12012 kb) Additional file 4:
**Normalization factors used in** ***egdeR.*** (XLSX 58 kb) Additional file 5:
**Differentially expressed gene models (DEGs).** (XLSX 1313 kb) Additional file 6:
**Description of statistical test.** (DOCX 89 kb) Additional file 7:
**Enriched GO terms.** (XLSX 203 kb) Additional file 8:
**Genes associated with response GO terms for upregulated DEGs in 592-roots and 520-roots.** (XLSX 72 kb)

## Abbreviations

ANOVA: Analysis of variance
bp: base pair
C ontology: Cellular component ontology
cm: Centimetre
DEG: Differentially expressed gene
df: Degrees of freedom
DW: Dry weight
F ontology: Molecular function ontology
FC: Fold change
FDR: False discovery rate
FPKM: fragments (read pairs) per kilobase of transcript per million mapped fragments
G x T: Genotype x Treatment interaction
GO: Gene ontology
h: hours
LAP: Leaf area productivity
LAR: Leaf area ratio
LW/W: Leaf biomass per total plant biomass
N: Nitrogen
*p*: *p*-value
P ontology: Biological process ontology
QTL: Quantitative trait locus
RGR: Relative growth rate
RNA-seq: RNA sequencing
RW/W: Root biomass per total plant biomass
RWC: Relative water content
SD: Standard deviation
SLA: Specific leaf area
SPAD: Leaf chlorophyll content
SW/W: Shoot biomass per total plant biomass
WW: Well-watered
Δ^13^C: Carbon isotope ratio ^13^C/^12^C
Δ^18^O: Oxygen isotope ratio ^18^O/^16^O
