# Erratum to: Genome-wide transcriptional and physiological responses to drought stress in leaves and roots of two willow genotypes

**DOI:** 10.1186/s12870-015-0665-4

**Published:** 2015-12-03

**Authors:** Pascal Pucholt, Per Sjödin, Martin Weih, Ann Christin Rönnberg-Wästljung, Sofia Berlin

**Affiliations:** Department of Plant Biology, Uppsala BioCenter, Linnean Centre for Plant Biology, Swedish University of Agricultural Sciences, Uppsala, SE-750 07 Sweden; Department of Evolutionary Biology, Evolutionary Biology Centre, Uppsala University, Uppsala, SE-752 36 Sweden; Department of Crop Production Ecology, Linnean Center for Plant Biology, Swedish University of Agricultural Sciences, Uppsala, SE-750 07 Sweden

## Erratum

Following publication of this work [1] it was noticed that an incorrect version of Fig. 1 was published. The figure contained 18 panels from A-U. However panel J was not listed and this should have read A-T. The correct version of Fig. 1 has been corrected in the original article and is also included correctly below. The publisher apologizes for any inconvenience caused.

**Fig. 1:**
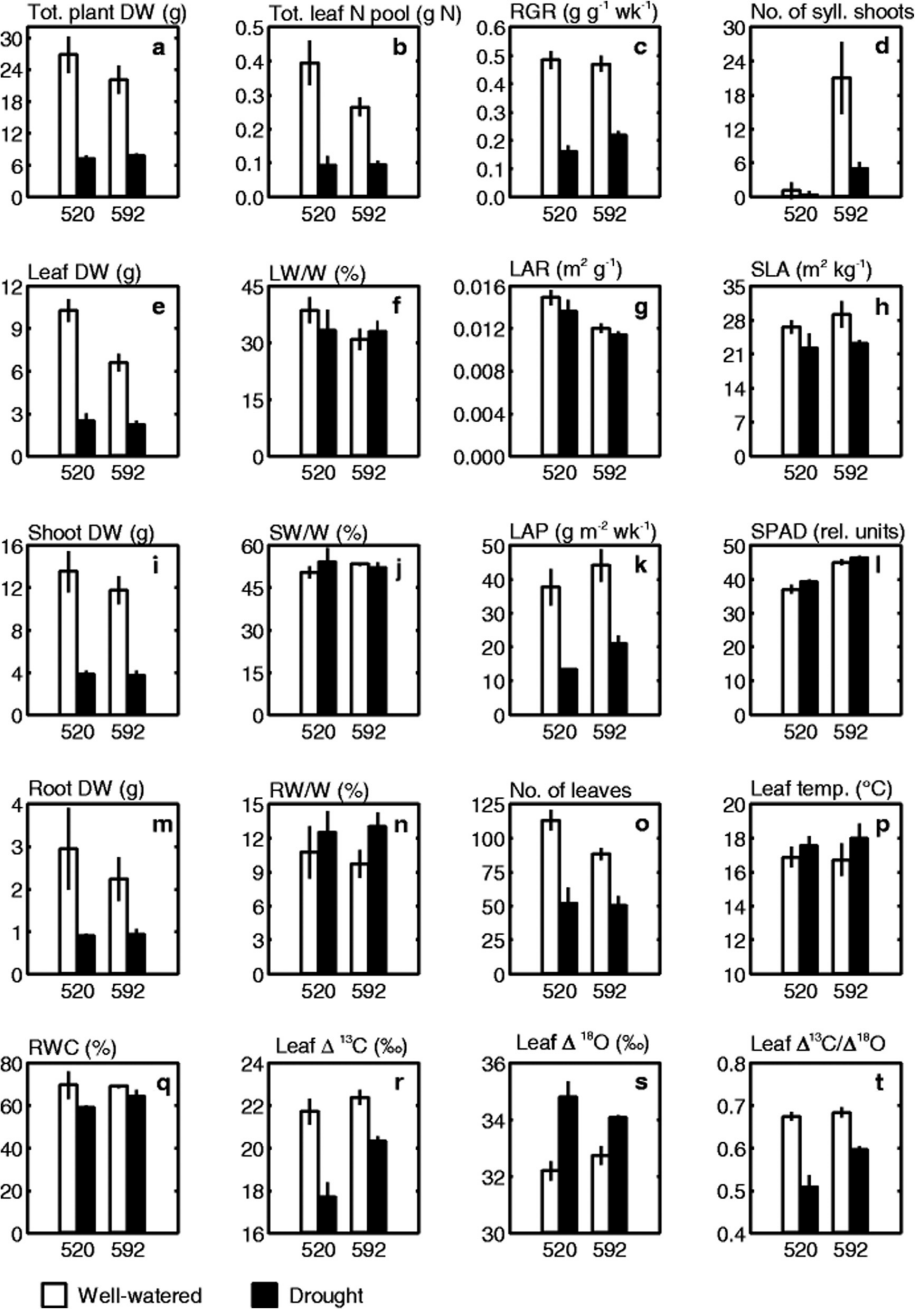
ᅟ
