# Supplementary material for: Genome-wide transcriptional and physiological responses to drought stress in leaves and roots of two willow genotypes
Source: BMC Plant Biol. 2015 Oct 12;15:244. doi: 10.1186/s12870-015-0630-2 (PMC4604075; doi:10.1186/s12870-015-0630-2)
Supplement: Additional file 6: — Description of statistical test. (DOCX 89 kb) [file 12870_2015_630_MOESM6_ESM.docx]

A reasonable way to measure the difference in drought response between the two genotypes 520 and 529 is to calculate

genResp=| log(foldchange(520)) – log(foldchange(592)) |

To calculate foldchange we used the sum of the raw counts divided by the effective library size in the well watered control samples (x0) and in the drought samples (x1). The foldchange was then calculated as x1/x0. In the cases when x1=0 and x0=0, foldchange was set to 1, when x1=0 and x0>0, we set foldchange to 10^(-8) and when x1>0 and x0=0, foldchange was set to 10^8.

The absolute value is used because we are not interested in whether a gene is up-regulated or down-regulated or which of the two genotypes reacted the strongest – just the difference in response between the genotypes.

This statistic was calculated separately for leaves (“genResp(leaf)”) and roots (“genResp(root)”) after which we counted how often genResp(leaf)>genResp(root). Among the 28 gene models, 25 gene models had a higher response in leaves than in roots.

To calculate a p-value, we assumed a null model with an equal chance of genResp(leaf)>genResp(root) as genResp(leaf)<genResp(root) among each of the 28 gene models with an FDR<0.05. Under this null model, to observe such a large deviance from the expected number of cases with genResp(leaf)>genResp(root) among 28 gene models (= 0.5*29) is calculated as

where Bin(i;29,0.5) is the binomial probability function
